# Co-spread of metal and antibiotic resistance within ST3-IncHI2 plasmids from *E. coli* isolates of food-producing animals

**DOI:** 10.1038/srep25312

**Published:** 2016-05-04

**Authors:** Liangxing Fang, Xingping Li, Liang Li, Shumin Li, Xiaoping Liao, Jian Sun, Yahong Liu

**Affiliations:** 1 National Risk Assessment Laboratory for Antimicrobial Resistance of Animal Original Bacteria, South China Agricultural University, Guangzhou, China; 2 Laboratory of Veterinary Pharmacology, College of Veterinary Medicine, South China Agricultural University, Guangzhou 510642, P. R. China; 3 Jiangsu Co-Innovation Centre for Prevention and Control of Important Animal Infectious Diseases and Zoonoses, Yangzhou, Jiangsu, the People’s Republic of China

## Abstract

Concerns have been raised in recent years regarding co-selection for antibiotic resistance among bacteria exposed to heavy metals, particularly copper and zinc, used as growth promoters for some livestock species. In this study, 25 IncHI2 plasmids harboring *oqxAB* (20/25)*/bla*_CTX-M_ (18/25) were found with sizes ranging from ∼260 to ∼350 kb and 22 belonged to the ST3-IncHI2 group. In addition to *bla*_CTX-M_ and *oqxAB*, *pcoA*-*E* (5/25) and *silE*-*P* (5/25), as well as *aac*(*6*′)*-Ib-cr* (18/25), *floR* (16/25), *rmtB* (6/25), *qnrS1*(3/25) and *fosA3* (2/25), were also identified on these IncHI2 plasmids. The plasmids carried *pco* and *sil* contributed to increasing in the MICs of CuSO_4_ and AgNO_3_. The genetic context surrounding the two operons was well conserved except some variations within the *pco* operon. The ~32 kb region containing the two operons identified in the IncHI2 plasmids was also found in chromosomes of different Enterobacteriaceae species. Further, phylogenetic analysis of this structure showed that Tn7-like transposon might play an important role in cross-genus transfer of the *sil* and *pco* operons among Enterobacteriaceae. In conclusion, co-existence of the *pco* and *sil* operons, and *oqxAB/bla*_CTX-M_ as well as other antibiotic resistance genes on IncHI2 plasmids may promote the development of multidrug-resistant bacteria.

The horizontal transfer of plasmids plays a significant role in the dissemination of antibiotic resistance genes. Plasmids in the HI incompatibility group (IncHI) occur widely in the Enterobacteriaceae. Members of this group can carry a wide variety of resistance genes including those encoding the metallo-β-lactamase NDM-1^1^,^2^. One subgroup of IncHI, IncHI2, is one of the most common incompatibility groups of plasmids in Enterobacteriaceae^3^. This group is frequently detected in *Salmonella enterica*, *Enterobacter cloacae*, *Klebsiella pneumonia* and *Escherichia coli* isolates from humans and chickens^4^,^5^,^6^, but also with a sporadic occurrence in swine^7^,^8^.

IncHI2 plasmids have been found to carry numerous classes of resistance genes including resistance to β-lactams (*bla*_CTX-M_, *bla*_CMY_, *bla*_SHV_, *bla*_IMP_, *bla*_VIM_), quinolones (*oqxAB*, *qnrA1*, *qnrS1* and *qnrB2*), aminoglycosides (*armA*, *aac-Ib*/*aac-Ib-cr*), amphenicols (*floR*) and fosfomycin (*fosA3*)^3^,^9^,^10^,^11^,^12^. Reports on co-spread of extended-spectrum β-lactamase (ESBLs) and plasmid-mediated quinolone resistance determinants (PMQRs) in the same plasmids have increased in the past years^6^,^13^. Our previous studies determined that IncHI2 plasmids are linked to the distribution of *oqxAB*-*bla*_CTX-M_ genes in *E. coli* and *Salmonella spp.*^10^,^11^. However, only a few of these cases have been documented. Fluoroquinolones such as ciprofloxacin and enrofloxacin, and cephalosporins such as ceftiofur have been widely used in veterinary medicine in China. Olaquindox, the main substrate for OqxAB, is also commonly used as a therapeutic and preventive antibiotic in pigs^14^.

In addition to genes encoding antibiotic resistance, the IncHI2 plasmids also harbor a large number of metal tolerance genes. For example, R478 is the prototype of the ST1-IncHI2 plasmids and has been totally sequenced^15^. It encodes efflux systems to detoxify copper (*pcoABCDRSE*), silver (*silESRCBAP*), arsenic (ars*CBRH*), as well as the Tn1696-related mercury operon (*merEDACPTR*) and tellurite resistance systems (*terZABCDEF* and *terY3Y2XY1W*). Moreover, trace elements including copper have been used as feed additives for the treatment of swine and poultry disease control and weight improvement^16^,^17^.

There is increasing concern that metal contamination functions as a selective agent in the proliferation of antibiotic resistance^18^. There is indeed experimental evidence that exposure to heavy metals (particularly copper and zinc) can induce or select for bacterial adaptations that result in decreased susceptibility to β-lactams^19^. This may occur by selection of heavy metal resistance determinants for resistance to non-antibiotic agents that are linked to genes for antibiotic resistance^20^. Considering that the IncHI2 plasmids may play an important role in dissemination of antibiotic and metal resistance genes, we characterized IncHI2 plasmids harboring *oqxAB* and/or *bla*_CTX-M_ in *E. coli* isolates from the diseased food-producing animals in China. Furthermore, the genetic context surrounding the *pco* and *sil* operons located on these IncHI2 plasmids were also investigated.

## Results

### The prevalence of the IncHI2 plasmids

Our initial study group contained 739 *E. coli* isolates from diseased animals. 405 of these isolates possessing either *bla*_CTX-M_ (204) or *oqxAB* (328) were selected for conjugation experiments. We were successful in obtaining 163 transconjugants harboring *bla*_CTX-M_ and/or *oqxAB*, including 25 that carried IncHI2 plasmids (25/163 total, 15.3%). The donor strains of these 25 transconjugants were isolated from 14 ducks, 4 chickens and 7 pigs among 2004–2012 and these food-producing animals were from 15 farms (Table 1).

### Detection of antimicrobial and heavy metal resistance determinants

Among the 25 transconjugants harboring IncHI2 plasmids, 20 carried *oqxAB*, and 17 harbored *bla*_CTX-M-9G_, while only one was positive for *bla*_CTX-M-1G_. The most predominant CTX-M-encoding gene was *bla*_CTX-M-14_ (6), followed by *bla*_CTX-M-27_ and *bla*_CTX-M-65_ (5 each). *OqxAB* and *bla*_CTX-M_ were found together in 13 transconjugants (Table 1). Other antibiotic-resistance determinants, *aac* (*6*′)*-Ib-cr*, *floR*, *qnrS1*, and *fosA3* were co-transferred in 18, 16, 3, and 2 transconjugants, respectively. The number of transconjugants carrying *oqxAB*-*aac*(*6*′)*-Ib-cr*, *oqxAB*-*floR*, and *oqxAB*-*aac*(*6*′)*-Ib-cr*-*floR*, were 15, 12, and 11, respectively. Moreover, four transconjugants carried *oqxAB*, *bla*_CTX-M-9G_ and *rmtB* simultaneously (Table 1). Interestingly, all of the 25 transconjugants carried a tellurite-resistance system while mercury and arsenic resistance genes were not detected. *PcoA*-*D*-*E*, as well as sil*E*-*P* genes was found in four transconjugants. Additionally, in one transconjugants S151T, *pcoA*-*E* was observed, while *pcoD* was not detected. (Table 1).

### Antimicrobial susceptibility tests

Among the 25 transconjugants harboring IncHI2 plasmids, 18 carried *bla*_CTX-M_ and showed a reduced susceptibility to CTX (MIC ≥2 μg/mL). In addition, 25 and 15 transconjugants were also resistant to AMP and CIF, respectively. At least one PMQR gene was found in 25 transconjugants (except S100T). Ciprofloxacin MICs were mainly grouped into two levels including 15 non-susceptible transconjugants (0.06–0.25 μg/mL) and 9 with low resistance levels (0.5–4 μg/mL). The MICs of OQX in 20 transconjugants carrying *oqxAB*, had 4-fold higher than that for the recipient *E. coli* C600. All transconjugants showed increase in MICs of FLF, and 11 showed extremely high-level resistance with MICs ≥256 μg/mL. Notably, co-transfer of extremely high-level resistance to AMK and FOS (MICs ≥256 μg/mL) were also observed in six transconjugants harboring *rmtB* and two carrying *fosA3*, respectively. None of the transconjugants were resistant to meropenem. The metal susceptibility testing showed that 5 transconjugants carrying the *pco* and *sil* genes had the MICs of CuSO_4_ and AgNO_3_ higher than that for the recipient *E. coli* C600 (MIC_CuSO_4__ = 12 mM vs. 8 mM; MIC_AgNO_3__ = 0.03~>1 mM vs. 0.008 mM), while in the other 20 of 25 transconjugants, the MICs of CuSO_4_ and AgNO_3_ had no change, when compared with *E. coli* C600 (Table 1).

### Plasmids analysis

The result of S1-PFGE revealed that all of the 25 transconjugants carried only one plasmid with size ranging from ~260 kb to ~380 kb, except for S151T which carried two plasmids (~260 kb and ~100 kb) (Table 1). Southern blot analysis confirmed that these large plasmids were members of the IncHI2 type. Furthermore, a probe hybridizing to *oqxB/bla*_CTX-M-9G_/*bla*_CTX-M-1G_/*rmtB*/*pcoA*/*silE* also confirmed that these genes were located on the IncHI2 plasmids. Interestingly, 16 of 25 (except pS151T) were fused plasmids. The most prevalent combination was IncHI2 in combination with IncFII (10) and followed by IncN (6) (Table 1). Using pDLST analysis, 22 IncHI2 plasmids were assigned to ST3 and only one to ST1 (pZ13T). Two IncHI2 plasmids were not typeable due to a failure to detect the smr0199 loci (pA84T and pS100T). RFLP analysis of plasmid DNA from the transconjugants harboring IncHI2 plasmids using *XbaI* demonstrated that 21 of 25 could be divided into eleven groups (designated A to K) (≥75% similarity) (Table 1).

The *hipA*, *mucB and relE* genes involved in plasmid stabilization were found in all of the 25 IncHI2 plasmids. However, the seven addiction systems tested in this study were completely lacking in eight plasmids containing only the IncHI2 replicon, as well as another four fused plasmids. Furthermore, no more than three addiction systems were detected among all of the 25 IncHI2 plasmids.

### Analysis of the genetic environment of the *oqxAB* and *bla*_*CTX-M*_ genes

The genetic environment of the *bla*_CTX-M-9G_ genes was IS*Ecp1*-*bla*_CTX-M-9G_-IS*903* (16), while it was IS*Ecp1*-*bla*_CTX-M-1G_-*orf477* for the *bla*_CTX-M-1G_ genes (1). In one transconjugants S7T, both the *bla*_CTX-M-9G_ allele and the genetic environment of the *bla*_CTX-M-9G_ gene were not determined. The *oqxAB* genes were flanked by two copies of IS26 that were located in the same orientation in 20 transconjugants harboring *oqxAB*. To determine the stability of this structure (IS26-*oqxA*-*oqxB*-IS26), inverse PCR was performed and amplicons of approximately 1.6 kb were obtained in all of 20 transconjugants. Sequence analysis of the amplicons further confirmed the genetic environment surrounding the *oqxAB* genes as obtained by PCR mapping.

### Analysis of the genetic environment of *pco* and *sil* genes

**Figure 1:**
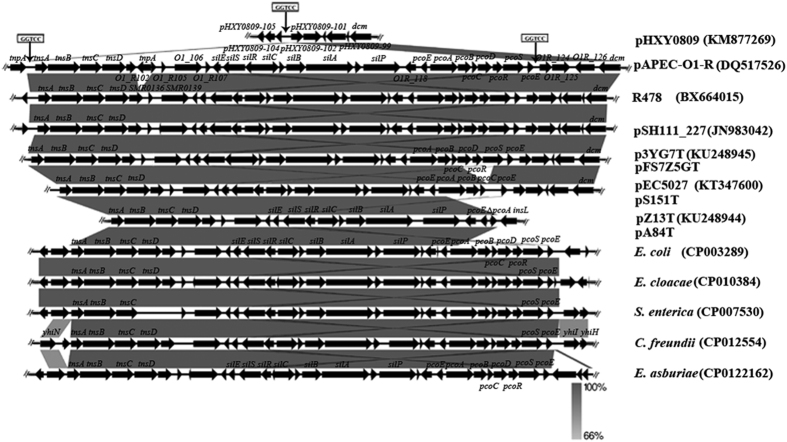
Characteristic of the genetic contexts of the *pco* and *sil* operons and linear comparison of the structures containing the *two* operons. The plasmid pHXY0809 (KM877269) represented an IncHI2 plasmid not carrying the *sil* and *pco* operons. Plasmids pAPEC-O1-R (BX663045), R478 (DQ517526), pSH111_227 (JN983042), and pEC5207 (KT347600) were the only four IncHI2 plasmids harbored the *sil* and *pco* operons assigned in GenBank. *E. coli* (CP003289), *E. cloacae* (CP010384), *S. enterica* (CP007530), *C. freundii* (CP012554), *E. asburiae* (CP0122162) represented the sequences containing the *sil* and *pco* operons and they were located on chromosomes of five different Enterobacteriaceae species. p3YG7T, pFS7Z5GT, pS151T, pZ13T, and pA84T represented the IncHI2 plasmids harbored the *sil* and *pco* operons in this study. The arrows represent the positions and transcriptional directions of the ORFs. Regions of homology are shaded in gray.

The regions surrounding the *pco* and *sil* genes are shown in Fig. 1, Supplementary Fig. S2 and Table S3. A Tn7-like transposon (~5.99 kb) encompassing the *tnsABCD* genes, and a ~4.64-kb region including four ORFs (encoding hypothetical proteins), were present upstream from the *sil* operon, which consisted of *silESRCBAP* genes (~12.45 kb). That was followed by a ~1.29-kb region including two ORFs (encoding hypothetical proteins). Downstream from it, three different genetic organizations were found within the *pco* operon: type I, in the plasmids p3YG7T and pFS7Z5GT, a ~7.53-kb segment containing the *pcoEABCDRSE* genes was present; type II, in the plasmid pS151T, the *pco* operon was identical to that in pEC5207 (KT347600) and the *pcoD* and *pcoR* genes were deleted; type III, in the plasmids pZ13T and pA84T, the *pco* operon was divided into two parts, and they were not genetically linked together: in one part, downstream from *pcoE*, *pcoA* had 1348 bp deleted at the 3′-end and then was followed by an insertion sequence *insL* (Fig. 1); in the other part, *pcoA* was truncated at the 5′-end by the insertion of *tnpA* in the reverse orientation, and *pcoBCDRSE* was present downstream (Supplementary Fig. S1). The *pco* operon was then followed by a 5.69-kb region including five ORFs in these five plasmids (Fig. 1 and Supplementary Fig. S1).

**Figure 2:**
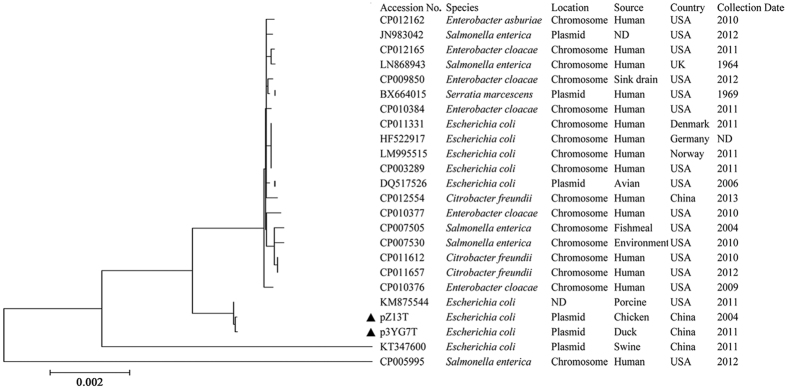
A phylogenetic analysis of *tnsABCD*-~4.64-kb region*-silESRCBAP*-~1.29-kb region-*pcoEABCDRSE* structure among 22 reference sequences from GenBank and two sequences pZ13T (KU248944) and p3YG7T(KU248945) (marked by the black triangles) in this study. The 22 reference sequences belonged to six different genera and were closely related to that in p3YG7T, with a 92–100% query coverage and an overall nucleotide identity of 99%. The GenBank accession number, the location of the *sil* and *pco* operons of each sequence and the host species, the sources, the locations of recovery and the collection dates of strains of each sequence are shown. The phylogenetic tree is constructed using MEGA 5.05 software.

To clarify the role of *tnsABCD*-~4.64-kb region*-silESRCBAP*-~1.29-kb region-*pcoEABCDRSE* (~32kb) in spread of the *sil* and *pco* operons, the similar regions from plasmids pZ13T (*tnsABCD*-~4.64-kb region*-silESRCBAP*-~1.29-kb region-*pcoE*-*∆pcoA*) and p3YG7T (*tnsABCD*-~4.64-kb region*-silESRCBAP*-~1.29-kb region-*pcoEABCDRSE*) were aligned with another 22 reference sequences downloaded from GenBank’s nucleotide database. The 22 reference sequences were closely related to that in p3YG7T, with a 92–100% query coverage and 99% overall nucleotide identity. A phylogenetic tree suggested that these regions were fairly conserved among the 24 selected sequences from chromosomes DNA or plasmids from isolates of six Enterobacteriaceae species. These isolates were recovered from six countries, and diverse origins including humans, animals and environment (Fig. 2).

## Discussion

In this study, 25 IncHI2 plasmids carrying *bla*_CTX-M_/*oqxAB* were found from 739 *E. coli* isolates from disease food-producing animals among 2004–2012. Additionally, we also found that *aac* (*6*′)-*Ib*-*cr* and *floR* were frequently co-transferred with *bla*_CTX-M_/*oqxAB* among these IncHI2 plasmids. *OqxAB* along with *aac* (*6*′)-*Ib*-*cr*, *floR*, and *bla*_CTX-M_ were identified on the same transferable IncHI2 plasmids^10^. This may be an important mechanism for dissemination of multidrug-resistance genes. Notably, *bla*_CTX-M_, *oqxAB*, and *rmtB* were the first time identified simultaneously on the IncHI2 plasmids from four transconjugants, which also showed different levels of MICs increased with CTX, CIP, and AMK as compared with recipient strain *E. coli* C600. The third-generation cephalosporin, fluoroquinolones, and aminoglycosides are the important front-line antibiotics. Therefore, these multidrug-resistant IncHI2 plasmids should be of great concern.

Insertion sequences IS*Ecp1* and IS*26* are most frequently associated with *bla*_CTX-M_ and *oqxAB*, respectively^11^,^21^. This is consistent with our results that IS*Ecp1* was upstream of the *bla*_CTX-M_ gene (except pS7T) and the *oqxAB* genes were flanked by IS*26* in this study. Interestingly, inverse PCR performed on all of the *oqxAB*-positive transconjugants produced an amplicon and subsequent sequencing showed that the pair of intact IS*26* flanking *oqxAB* could loop out the intervening sequence through homologous recombination. This might further accelerate *oqxAB* dissemination among Enterobacteriaceae. Further studies are required to explore how the diverse resistance genes, especially *bla*_CTX-M_ and *oqxAB*, as well as *rmtB*, were integrated into the same IncHI2 plasmids.

IncHI2 plasmids are high molecular weight and possess multiple replicons (RepHI1A and RepHI2)^15^. Additionally, the IncHI2 and IncFIB plasmids co-resident within the same cell were found to undergo plasmid fusion in the transconjugants^22^. In this study, the majority of the plasmids contained IncHI2 were in combination with either IncFII or IncN. Plasmid may have recombined with co-resident plasmids^23^, thereby expanding the number of replicons and extending host ranges of the fused plasmids. Although the IncHI2 plasmids herein were of diverse sizes, 22 of 25 were assigned to the ST3 group by pDLST. In Europe and the USA, *bla*_CTX-M-2_ producers from both human and poultry sources have been associated with ST2-IncHI2 plasmids, while the *bla*_CTX-M-9_ producers were associated with ST1-IncHI2 plasmids^4^. In China, ST3-IncHI2 plasmids have been found to spread *fosA3* among *E. coli* isolates from chickens^12^. This indicates that ST3-IncHI2 plasmids most often associated with resistance genes in *E. coli* from food-producing animals in China. Further, a variety of plasmid patterns were observed by comparing the similarity of the 25 IncHI2 plasmids using RFLP analysis, although some ones showed similar *XbaI* digestion profiles. Considering that IncHI2 plasmids possessed a well-conserved and stable backbones^3^, their diversity observed herein were probably due to the deletions or acquisition of a number of resistance genes by transposons and insertion sequences^24^ or IS26-mediated fusion with other plasmids^23^.

The presence of addiction systems encoded in resistance plasmids may allow for the maintenance and dissemination of resistance genes within a given bacterial population^25^. However, in this study, IncHI2 plasmids were found mostly to be devoid of addiction systems which had been previously shown^12^. These results are not surprising because the seven addiction systems detected in this and that studies were mainly characterized in IncF, IncI1 plasmids or *Salmonella* virulence plasmids^25^. However, *hipA/B*, *mucA/B*, *relE/B*, and *ter* determinants involved in plasmid stabilization system were observed among all of the 25 IncHI2 plasmids. This once again suggested that these genes might play a significant role in the persistence and spread of IncHI2 plasmids.

It has been known for several decades that metal- and antibiotic-resistance genes are linked, particularly on plasmids^26^,^27^. The *ter* determinants were found on the IncHI2 plasmids in the previous study^28^ and were also observed on all of the IncHI2 plasmids in this study. However, the *mer and ars* determinants were not found on any of the 25 IncHI2 plasmids. The prototype of the ST1-HI2 group, R478, harbors the *mer and ars* determinants, while they are deleted in pAPEC-O1-R, the prototype of ST2-HI2 plasmids^3^,^15^,^29^. The IncHI2 plasmids presented in this study may be genetically distinct from R478. Five of the 25 IncHI2 plasmids (20%) also harbored the *pco* and *sil* genes, simultaneously. The metal susceptibility testing showed that they also contributed to increasing in the MICs of CuSO_4_ and AgNO_3_ when compared with the recipient *E. coli* C600 and other transconjugants not carried the *pco* and *sil* genes. Surprisingly, there were some differences between the MICs of AgNO_3_, but the MICs of CuSO_4_ were identical in the five transconjugants carrying *pco* and *sil* genes.

We further analyzed the genetic background surrounding the *pco* and *sil* genes. The results indicated that the ~24-kb structures (*tnsABCD*-~4.64-kbregion*-silESRCBAP*-~1.29-kb region) and the 5.69-kb regions including five ORFs downstream from the *pco* operons were well conserved in the five plasmids in this study and another four IncHI2 plasmids (R478 (DQ517526), pSH111-27 (JN983042), pAPEC-O1-R (BX663045) and pEC5027 (KT347600)) from GenBank (Fig. 1). The *silESRCBAP* genes constituted the complete *sil* operon and shared 95~96.3% identity to that of plasmid pMG101 (AF067954) (Fig. S3A) which played a function role in conferring to silver resistance^30^. However, there was variability within the *pco* operon. In the plasmids p3YG7T and pFS7Z5GT, the complete *pco* operon composed of *pcoEABCDRSE* genes, was 99.4% identical to that of the plasmid PRJ1004 (X83541) (Fig. S3B) which facilitates copper efflux^31^. However, in another three IncHI2 plasmids pS151T, pZ13T and pA84T, the *pco* operons were disrupted even though they also shared high similarities with that of plasmid PRJ1004 (Fig. S3B). The deletion of the *pcoD* and *pcoR* genes was also observed in the IncHI2 plasmid pEC5027 carrying *bla*_CMY-2_ in our previous report^32^. It has been demonstrated that mutations in each of the *pcoABCD* genes on the plasmid pRJ1004 and the *silECBA* genes on plasmid pMG101 lead to complete loss of copper and silver resistance, respectively^31^,^33^. Thus, the reason for the inconsistency between the genetic contexts of the *pco* and *sil* operons and the MICs of CuSO_4_ and AgNO_3_ observed in the five transconjugants harboring *pco* and *sil* is unknown. This remains to be elucidated through further studies.

A Tn7-like transposon carried the *pco* and *sil* genes in IncHI plasmids from previous reports^33^,^34^. In the current study, aTn7-like transposon was also present upstream of the *pco* and *sil* operons in the five IncHI2 plasmids. In our previous study, we obtained the complete sequence of the IncHI2 plasmid pHXY0809 (KM877269) which carried *oqxAB* but did not harbor the *sil* and *pco* operons. Interestingly, a linear comparison of plasmid pHXY0809 with plasmids pAPEC-O1-R, R478, pSH111-27, pEC0527, and p3YG7T (this study) revealed that the regions containing *sil* and *pco* operons appeared to be mobilized into these IncHI2 plasmids via the Tn7-based transpositions (Fig. 1). We also identified a complete transposition unit flanked by 5 bp direct repeats (DR) (GTCCT) that bounded the *tnsABCD*-~4.64-kb region*-silESRCBAP*-~1.29-kb region*-pcoEABCDRSE* structure in the plasmid p3YG7T. Furthermore, a transposition unit containing the *sil* and *pco* operons, flanked by 5-bp DR (GGTCC or GTCCT), was also found in plasmids R478, pSH111-27, pAPEC-O1-R and pEC0527. These transposition units were all bordered by a 28 bp sequence (TGTCCGAGGACAATAAAGTTGTACACAA) at one end, and another 28 bp sequence (AAGGATACAACTTTAATGTCTCTACACA) at the other end. The two 28 bp sequences show 18-bp nucleotide identity. As Tn7 carries terminal inverted repeats of 28 bp^35^, we speculated that the two 28 bp sequences might serve as the invert repeats of Tn7-like transposons. These results revealed that Tn7-based transpositions may play a significant role in the spread of the *sil* and *pco* operons among IncHI2 plasmids.

A chromosomal integration of Tn7-like transposons carrying the *pco* and *sil* genes was also identified in *Salmonella* Senftenberg^34^. Tn7-based transposition appeared to be able to mobilize the *sil* and *pco* operons from plasmid into chromosome^33^. Interestingly, in the IncHI2 plasmids (R478, pSH111-27, and p3YG7T), the structures (*tnsABCD*-~4.64-kb region*-silESRCBAP*-~1.29-kb region-*pcoEABCDRSE*) were highly similar to that in chromosomes of five different genera (Fig. 1). This may implicate mobilization of the *sil* and *pco* operons from plasmids into chromosomes or conversely, from the chromosomes into plasmids via Tn7-based transposition. Further, phylogenetic analysis of this structure suggested that a Tn7-like transposon was involved in cross-genus transfer of the *sil* and *pco* operons among Enterobacteriaceae of diverse origins in many countries.

Copper has been commonly used as a feed additive in animal growth promotion as described above. Silver, on the other hand, is widely used in disinfectants during production or as animal antiseptics^36^,^37^. There is a strong association of heavy metal micronutrients in swine feed and the occurrence and persistence of multidrug-resistant bacteria^38^. Therefore, metal contamination may contribute to the persistence of the genetic platforms that carry metal and antibiotic resistance genes. These platforms include the IncHI2 plasmids and Tn7-like transposons which may serve to maintain and spread heavy metal-tolerant and multidrug-resistant Enterobacteriaceae.

In conclusion, we characterized 25 IncHI2 plasmids harboring *bla*_CTX-M_/*oqxAB* from *E. coli* isolates from diseased farm animals in China among 2002–2012. Co-spread of *bla*_CTX-M_/*oqxAB* with *aac* (*6*′)*-Ib-cr*, *floR*, *fosA3* and *rmtB*, as well as the heavy metal resistance genes (*pco* and *sil*), were identified on the large and diverse ST3-IncHI2 plasmids. These IncHI2 plasmids carried the *pco* and *sil* operons also contributed to increasing in the MICs of CuSO_4_ and AgNO_3_. Further, IS*Ecp1* and IS*26* were found to involve in spread of *bla*_CTX-M_ and *oqxAB*, respectively. Tn7-like transposons were linked to dissemination of the *sil* and *pco* operons. This is the first report of co-existence of *oqxAB*, *bla*_CTX-M_, and the *pco* and *sil* operons on the same plasmids. This may promote the dissemination of multidrug-resistant isolates under the metal and antibiotic selective pressure. Increased surveillance of the multidrug-resistant IncHI2 plasmids in *E. coli* food-producing animals is urgently needed.

## Materials and Methods

### Bacterial strains

A total of non-duplicate 739 *E. coli* strains were isolated from viscera or feces samples from diseased food-producing animals, including ducks (203), chickens (110), geese (31) and pigs (395) between 2002 and 2012 as described previously^10^,^39^. The samples were recovered from more than 80 livestock farms throughout Guangdong province. *E. coli* isolates carrying the *bla*_CTX-M_ and/or *oqxAB* genes (405/739) were selected in conjugation experiments by the broth-mating method using *E. coli* C600 (streptomycin-resistant; MIC >2000 μg/mL) as the recipient. The transconjugants were selected on MacConkey agar plates supplemented with streptomycin (500~1000 μg/mL) and cefotaxime (2 mg/L) or olaquindox (32~64 mg/L). The plasmids isolated from the transconjugants harboring *bla*_CTX-M_ and/or *oqxAB* were further characterized by PCR-based replicon typing (PBRT) using PCR amplification/sequencing with IncHI2 primers as previously described^40^.

### Antimicrobial susceptibility tests

For all of the transconjugants harboring IncHI2 plasmids, MICs of ampicillin (AMP), cefoxitin (FOX), ceftiofur (CIF), cefotaxime (CTX), amikacin (AMK), gentamicin (GEN), chloramphenicol (CHL), florfenicol (FLF), doxycycline (DOX), nalidixic acids (NAL), ciprofloxacin (CIP), olaquindox (OQX), sulfamethoxazole/trimethoprim (SXT), meropenem (MEO) were determined by the agar dilution method following the guidelines of Clinical and Laboratory Standards institute (CLSI). MIC of fosfomycin (FOS) was determined by the agar dilution method on Mueller-Hinton agar containing 25 μg/mL glucose 6-phosphate, according to guideline M100-S20 of the CLSI. The breakpoints for each antimicrobial were used as recommended by the CLSI (M100-S25) or CLSI (Vet01-A4/Vet01-S2)^41^,^42^. *E. coli* ATCC 25922 was used as a quality control strain. MICs of AgNO_3_ and CuSO_4_ were determined by broth microdilution method in an aerobic atmosphere as previously described^33^, with some modification. Briefly, the transconjugants harboring IncHI2 plasmids were incubated in Mueller-Hinton broth with serial dilutions of CuSO_4_ (0.25, 0.5, 1, 2, 4, 8, 12, 16, 20, 24, 32 and 36 mM, adjusted to pH 7.2) and AgNO_3_ (0.0004, 0.0008, 0.0015, 0.03, 0.06, 0.08, 0.125, 0.16, 0.25, 0.32, 0.5, 1.0 mM, adjusted to pH 7.4). *E. coli* C600 was used as a reference strain.

### Detection of antimicrobial and heavy metal resistance determinants

ESBL-encoding genes (*bla*_TEM_, *bla*_SHV_, *bla*_C*TX*-M-1G_, *bla*_CTX-M-9G_, *bla*_CTX-M-2G_, and *bla*_CTX-M-25G_), pAmpCs-encoding genes (*bla*_CMY-2_), PMQR genes (*qnrA*, *qnrB*, *qnrS*, *aac*-(*6*′)*-Ib-cr*, *qepA*, *oqxA* and *oqxB*), exogenously acquired 16S rRNA methyltransferase (16S-RMTase) genes (*rmtB* and *armA*), fosfomycin resistance genes (*fosA3*, *fosA*, and *fosC2*) and the florfenicol resistance gene (*floR*) were detected among all of the transconjugants harboring IncHI2 plasmids by PCR amplification using primers published previously^9^,^10^,^12^,^43^,^44^. Metal resistance determinants, including *terD*, *terF*, *terX* and *terY3* (conferring resistance to tellurium), *merA* and *merC* (conferring resistance to mercury), *arsB* and *arsH* (conferring resistance to arsenic), *pcoA*, *pcoD* and *pcoE* (conferring resistance to copper), *silE* and *silP* (conferring resistance to silver) were also detected among these transconjugants by PCR amplification (Table S1).

### Plasmids analysis

Plasmids analysis was carried out in the transconjugants harboring IncHI2 plasmids by DNA linearization with S1 nuclease followed by PFGE analysis^45^. *Salmonella enterica* serotype Braenderup H9812 standards and Lambda Ladder PFG marker (NEB, Biolabs) were used as size markers. Southern blotting was carried out on S1-PFGE gels with digoxigenin-labelled probes specific for the IncHI2 replicon, *oqxB, bla*_CTX-M-9G_, *bla*_CTX-M-1G_, *rmtB*, *pcoA and silE*. Incompatibility (Inc) groups were assigned by PBRT of the transconjugants^40^. Plasmid double-locus sequence typing (pDLST) for IncHI2 plasmids was performed as previously described^3^. The IncHI2 plasmids were further analyzed by restriction fragment length polymorphism (RFLP) using *Xba*I as the restriction enzymes (TaKaRa Biotechnology, Dalian, China). Comparison of RFLP patterns was performed with BioNumerics^®^v6.6 (Applied Maths, Ghent, Belgium). Dendrograms were generated with the Dice similarity coefficient (1.5% optimization and 1.5% tolerance) using the unweighted pair group method with arithmetic mean. RFLP types were defined with ≥75% similarity between clusters. Additionally, to further understand the successful dissemination of the IncHI2 plasmids, plasmid addiction systems were determined^25^ and another three genes *hipA*, *mucB* and *relE* involving in plasmid stabilization system were also detected (Table S1).

### Analysis of the genetic environment of resistance genes

The genetic context surrounding *oqxAB* and *bla*_CTX-M_ on the IncHI2 plasmids were investigated by PCR mapping, inverse PCR and sequencing. The primers used to determine the regions upstream and downstream of the *oqxAB* and *bla*_CTX-M_ genes are listed in Table S1. The genetic contexts of *pco* and *sil* genes on the IncHI2 plasmids were also explored by PCR mapping and primer walking. The region containing the *pco* and *sil* genes in plasmids pEC5207 (KT347600) was using as the reference sequence (Supplementary Table S2).

### Nucleotide Sequence Accession Numbers

The two partial nucleotide sequences of plasmid pZ13T containing the *sil* operon and the *pcoBCDRSE* genes have been deposited into GenBank under accession numbers KU248944 and KU248943, respectively. The partial nucleotide sequences of plasmid p3YG7T containing the *sil* and *pco* operons has also been deposited into GenBank under accession numbers KU248945.

## Additional Information

**How to cite this article**: Fang, L. *et al.* Co-spread of metal and antibiotic resistance within ST3-IncHI2 plasmids from *E. coli* isolates of food-producing animals. *Sci. Rep.* **6**, 25312; doi: 10.1038/srep25312 (2016).

## Supplementary Material

Supplementary Information

## Figures and Tables

**Table 1:** Characteristics of the 25 *E. coli* isolates and transconjugants harboring IncHI2 plasmids.

Strain	Source	Farm no.	Year	Co-transferred resistance genes				MICs (ug/ml)/(mM)				Plasmid			
				ESBLs	PMQRs	Metal resistance genes	Other genes	CTX	CIP	CuSO_4_	AgNO_3_	Replicon types	Size (kb)	Addiction system	*X-baI*RFLP
Z39	Chicken	Farm 1	2004	*bla*_CTX-M-27_	*oqxAB, aac*(*6*′)*-Ib-cr*	–	*rmtB, floR*	8	0.25	8	0.008	HI2, FII	∼350	*hok-sok*, *pemKI*, *srnBC*	G3
Z13	Chicken	Farm 1	2004	*–*	*oqxAB*	*pcoA*-*D*-*E*, *silE-P*	*rmtB*	0.06	0.06	12	0.03	HI2, FII	∼350	*hok-sok*	NT
Z31	Chicken	Farm 1	2004	*–*	*oqxAB*	*–*	*–*	0.06	0.06	8	0.008	HI2, FII	∼350	*hok-sok*, *srnBC*, *ccdAB*	F4
S7	Pig	Farm 2	2004	*bla*_CTX-M-9G_	*oqxAB, aac*(*6*′)*-Ib-cr*	*–*	*floR*	4	0.5	8	0.008	HI2	∼280	no	I
X2	Duck	Farm 3	2005	*bla*_CTX-M-65_	*oqxAB, aac*(*6*′)*-Ib-cr*	*–*	*floR*	32	1	8	0.008	HI2, N	∼280	no	C
A84	Duck	Farm 4	2005	*–*	*oqxAB*	*pcoA*-*D*-*E*, *silE-P*	*rmtB*	0.25	0.125	12	0.06	HI2, FII	∼280	*hok-sok*	G2
A64	Duck	Farm 5	2007	*bla*_CTX-M-27_	*oqxAB, aac*(*6*′)*-Ib-cr*	*–*	*rmtB, floR*	32	0.25	8	0.008	HI2, FII	∼350	*hok-sok*	NT
A69	Duck	Farm 5	2007	*bla*_CTX-M-27_	*oqxAB, aac*(*6*′)*-Ib-cr*	*–*	*rmtB, floR*	32	0.125	8	0.008	HI2, FII	∼350	*hok-sok*	E
A74	Duck	Farm 5	2007	*bla*_CTX-M-27_	*oqxAB, aac*(*6*′)*-Ib-cr*	*–*	*rmtB, floR*	32	0.5	8	0.008	HI2, FII	∼320	*hok-sok*	NT
A78	Duck	Farm 5	2007	*bla*_CTX-M-27_	*oqxAB, aac*(*6*′)*-Ib-cr*	*–*	*–*	32	0.25	8	0.008	HI2, N	∼280	*no*	G1
S100	Duck	Farm 6	2007	*bla*_CTX-M-14_	*–*	*–*	*floR*	32	0.03	8	0.008	HI2, FII, N	∼280	*hok-sok*	H
S151	Duck	Farm 6	2007	*–*	*oqxAB, aac*(*6*′)*-Ib-cr*	*pcoA*-*E*, *silE-P*	*–*	0.06	0.06	12	>1	HI2, FII	∼260/100	*hok-sok*	D2
P2-3	Pig	Farm 7	2008	*bla*_CTX-M-65_	*oqxAB, aac*(*6*′)*-Ib-cr*	*–*	*floR*, *fosA3*	32	0.5	8	0.008	HI2, FIB	∼380	*hok-sok*, *pemKI*	F2
P3-3	Pig	Farm 7	2008	*–*	*oqxAB*	*–*	*–*	8	0.25	8	0.008	HI2, FII, FIB	∼260	no	F1
HAI	Pig	Farm 8	2009	*–*	*oqxAB, aac*(*6*′)*-Ib-cr*	*–*	*–*	0.06	0.25	8	0.008	HI2	∼280	no	K2
FS341G	Duck	Farm 9	2010	*bla*_CTX-M-65_	*qnrS1*, *aac*(*6*′)*-Ib-cr*	*–*	*floR*	32	0.5	8	0.008	HI2, N	∼280	VagCD	A
45-6	Pig	Farm 10	2010	*bla*_CTX-M-14_	*oqxAB*, *aac*(*6*′)*-Ib-cr*	*–*	*floR*	32	0.125	8	0.008	HI2	∼280	no	J2
FS271X	Duck	Farm 9	2010	*bla*_CTX-M-65_	*aac*(*6*′)*-Ib-cr*	*–*	*floR*	32	0.125	8	0.008	HI2, N	∼280	no	J3
2Y4G	Duck	Farm 11	2011	*bla*_CTX-M-14_	*oqxAB*, *aac*(*6*′)*-Ib-cr*	*–*	*floR*	2	0.25	8	0.008	HI2	∼280	no	B
3YG7	Duck	Farm 11	2011	*–*	*oqxAB, aac*(*6*′)*-Ib-cr*	*pcoA*-*D*-*E*, *silE-P*	*floR*	0.06	0.5	12	>1	HI2	∼280	no	D3
CBJ3C	Chicken	Farm 12	2012	*bla*_CTX-M-14_	*oqxAB*	*–*	*floR, foSA3*	16	0.06	8	0.008	HI2, N	∼350	*PemKI*, *srnBC*	NT
FS8Z4C	Pig	Farm 13	2012	*bla*_CTX-M-65_	*qnrS1*	*–*	*floR*	32	2	8	0.008	HI2, FII	∼280	*hok-sok*	F3
FS1Z4S	Pig	Farm 14	2012	*bla*_CTX-M-14_	*oqxAB, aac*(*6*′)*-Ib-cr*	*–*	*floR*	8	0.125	8	0.008	HI2	∼280	no	K1
FS7Z5G	Pig	Farm 13	2012	*bla*_CTX-M-14_	*aac*(*6*′)*-Ib-cr*	*pcoA*-*D*-*E*, *silE-P*	*–*	32	0.5	12	>1	HI2,	∼280	no	D1
FS11Y5C	Duck	Farm 15	2012	*bla*_CTX-M-55_	*oqxAB, aac*(*6*′)*-Ib-cr*, *qnrS1 aac*(*6*′)*-Ib-cr qnrS1*	*–*	*–*	64	4	8	0.008	HI2	∼260	no	J1

CTX, cefotaxime; CIP, ciprofloxacin; “–” “not detected”; “NT” “not determined”.
