# Supplementary material for: Co-spread of metal and antibiotic resistance within ST3-IncHI2 plasmids from E. coli isolates of food-producing animals
Source: Sci Rep. 2016 May 4;6:25312. doi: 10.1038/srep25312 (PMC4855149; doi:10.1038/srep25312)
Supplement: Supplementary Information [file srep25312-s1.pdf]

Co-spread of metal and antibiotic resistance within ST3-IncHI2 plasmids from *E. coli* isolates of food-producing animals

Liangxing Fang[1,2], Xingping Li[1,2], Liang Li[1, 2], Shumin Li[1, 2], Xiaoping Liao[1, 2], Jian Sun[1,2] & Yahong Liu[1, 2, 3]

[1]National Risk Assessment Laboratory for Antimicrobial Resistance of Animal Original Bacteria, South China Agricultural University, Guangzhou, China. [2]Laboratory of Veterinary Pharmacology, College of Veterinary Medicine, South China Agricultural University, Guangzhou 510642, P. R. China. [3]Jiangsu Co-Innovation Centre for Prevention and Control of Important Animal Infectious Diseases and Zoonoses, Yangzhou, Jiangsu, the People's Republic of China. Correspondence and requests for materials should be addressed to J.S. or Y.L. (email: jiansun@scau.edu.cn or lyh@scau.edu.cn).

Table S1 Primers used for PCR and sequence analysis

PCR	Primer[a]	Primer sequence (5' → 3')	PCR product length(bp)	Target	Reference
A	A[F]	CCACTGCGCGGAATTTCCACTCACCAT	231	screening for *terD*	(1)
	A[R]	ACGCCGTCCCGTCTGATGTTGACAAG			
B	B[F]	CCGACAAACTTCCAGAAGATGGGGTAGT	428	screening for *terF*	(1)
	B[R]	GAGGCAGCGGTTGCATTTGTACTTGACG			
C	C[F]	ATGCGCCGCCTGCCTGTTTACCTTGTTA	576	screening for *terX*	(1)
	C[R]	CGCGCTTGTGCTGCCGGAAGACA			
D	D[F]	CCTGGGGCCGTCAGCGGACCTG	302	screening for *terY3*	(1)
	D[R]	TCCTTGCTGGTGGCCGTTCATACTTCAT			
E	E[F]	CATGCCCTTGTCGATGACGAT	575	screening for *merA*	This study

	E^R	GCAATTCAGCCATCACCGTCC			
F	F^F	GGCCGACATAGAGCAGTCCAC	313	screening for *merC*	This study
	F^R	CGCATCGCTGGCAAAACCG			
G	G^F	AGTGAAAGACAGACGAAGCG	244	screening for *arsB*	(2)
	G^R	GGCAGATAGTGTGGAATGCG			
H	H^F	GAAGGACGAACAGCACCTTC	276	screening for *arsH*	(3)
	H^R	CGCATCTGATTCACAGCATT			
I	I^F	ATCCGGAAGGTCAGCACCGTCCATAGAC	507	screening for *pcoA*	(1)
	I^R	GACCTCGCGGATGTCAGTGGCTACACCT			
J	J^F	GGCGCCCAGAATGATAATCGCAACA	502	screening for *pcoD*	(1)
	J^R	GGGCGTGGCGCTGGCTACACTT			

K	K^F	GTGGGGCAGCTTTTGCTCAGTCCAGTGA	385	screening for *pcoE*	(1)
	K^R	CGAAGCTTTCTTGCCTGCGTCTGATGTG			
L	L^F	TCGGCCTGGGCCACTGAAACCGTGAATA	364	screening for *silE*	(1)
	L^R	GGCGGTGCGCTTCGGCCATAGCCTGATG			
M	M^F	ACACCCCGGCCTGGGCTCCTT	603	screening for *silP*	(1)
	M^R	TGCGGGCACGGGAACAAACCTC			
N	N^F	AAATGAATGGCTCTGCTCGGTA	585	screening for *hipA*	This study
	N^R	ATTTCCTCTATCATCGCCTCT			
O	O^F	CCGCCTCGCTATATACTTCCG	240	screening for *relE*	This study
	O^R	CACTAAAGGAATGGCGAAAGCTG			
P	P^F	TTGATTCAGCAACACCGGAT	597	screening for *mucB*	This study
	P^R	TATCTCATTAAGGCGCTGACC			
Q	Q^F	CTATCCGTACAAGGGAGTGT	～1600	junction between the 5’ end of	(4)

	Q^{R}	TTTCCACTCGCCTTCACC		IS*Ecp1* and 3’ end of IS903	
R	R^{F}	CTATCCGTACAAGGGAGTGT	～1300	junction between the 5’ end of	(4)
	R^{R}	CAGCGGAAGGAGAACCAG		IS*Ecp1* and 3’ end of *orf477*	(4)
S	S^{F}	ATGAACCCATTCAAAGGCCG	～4500	junction between the 5’ end of	(5) (6)
	S^{R}	GCGGTGCTGTCGATTTTA		IS*26* and 5’ end of *oqxB*	
T	T^{F}	TTACATTTCAAAAACTCTGCTTACC	～3000	junction between the 3’end of IS*26*	(5) (6)
	T^{R}	TACCGGAACCCATCTCGAT		and 3’ end of *oqxB*	
U	U^{F}	CCTGCCGAATGGGATGAAT	～1800	inverse PCR amplication of the	This study
	U^{R}	CGCTTCAATGCGACCGTTA		IS*26*-*oqxAB*- IS*26* region	

[a]F, forward primer; R, reverse primer.

References

[1] Johnson TJ, Wannemeuhler YM, Scaccianoce JA, Johnson SJ, & Nolan LK. Complete DNA sequence comparative genomics, and prevalence of an IncHI2 plasmid occurring among extraintestinal pathogenic *Escherichia coli* isolates. *Antimicrob Agents Chemother*. **50,** 3929-3933 (2006)

[2] Garcia Fernandez, A. *et al*. Comparative analysis of IncHI2 plasmids carrying *bla*$_{CTX-M-2}$ or *bla*$_{CTX-M-9}$ from *Escherichia coli* and *Salmonella enterica* strains isolated from poultry and humans. *Antimicrob Agents Chemother*. **51**, 4177-4180 (2007).

[3] Ryan D, Colleran E. Arsenical resistance in the IncHI2 plasmids. *Plasmid*.**47,** 234-240 (2002).

[4] Sun Y, Zeng Z, Chen S, Ma J, He L, et al. High prevalence of *bla*$_{(CTX-M)}$ extended-spectrum beta-lactamase genes in *Escherichia coli* isolates from pets and emergence of CTX-M-64 in China. *Clin Microbiol Infect*. **16,** 1475-1481 (2010).

[5] Rodriguez-Martinez JM, Diaz de Alba P, Briales A, Machuca J, Lossa M, et al. Contribution of OqxAB efflux pumps to quinolone resistance in extended-spectrum-beta-lactamase-producing *Klebsiella pneumoniae*. *J Antimicrob Chemother*. **68,** 68-73 (2013)

[6]Wang X-M, Liao X-P, Sun J, Zhu H-Q, Chen X-Y, et al. Plasmid-mediated quinolone resistance determinants *oqxAB* and aac(6')-Ib-cr and extended-spectrum beta-lactamase gene *bla*$_{CTX-M-24}$ co-located on the same plasmid in one *Escherichia coli* strain from China. *J Antimicrob Chemother*. **66,** 1638-1639 (2011).

Table S2 Primers used for PCR and sequence analysis

Primer	Sequence (5'to 3', as synthesized)	Expected amplicon size (bp)	Target	Nucleotide positions	GenBank accession no.[a]
A-1F	CGGCTTACCCAACAAATAGCAA	6564	junction between *tnsA* and conserved hypothetical protein	161249-161270	KT347600
A-1R	ATCATCAAGTCCGATAGAGGCAT			154707-154729	
B-2F	GATTATCATTACTGGGAGCCTA	6943	junction between *tnsB* and *cusS* (*silS*)	156325-156346	KT347600
B-2R	CACCTCCGAGAATCTTGATGC			149404-149424	
C-3F	GAAACGGTCTGACTTCCCAT	5499	junction between *silE* and *silB*	150729-150748	KT347600
C-3R	CTGAATCTGAGCCACCACCT			145249-145268	
D-4F	CTATTCCCGACAGGTTAACGAA	8409	junction between *silC* and cell wall endopeptidase	146527-146548	KT347600
D-4R	CCCGCTGATAACAAACTCGAAC			138098-138119	
E-5F	GGATAACGCAATAGCACCTCC	3668	junction between cell wall endopeptidase and *copB*(*pcoB*)	137984-138004	KT347600
E-5R	CAGTTGATCCAGCAGAACCAG			134337-134357	
F-6F	CGTATCACTGCCATTTGCTCT	4925(2644)	junction between *copA* (*pcoA*)and *copE* (*pcoE*)	134686-134709	KT347600
F-6R	CATTTCAGCCATGTCCTGGTG			132066-132086	
G-7F	CAGTCTTCCGTTGTTCACCA	5656	junction between *pcoE* and *dcm*	131932-131951	KT347600
G-7R	ACAGCTTCGACCATTTATGACC			126294-12315	

H-8F	ATTACGTGCCGAAGTATCCAG	5588	junction between *tnpA* and *copE*	1265-1285	KU248943
H-8R	TTATGCATTTCAGCCATGTCC			7606-7626	
I-9F	CTGGCCCCTTTAGTAACGAG	3055	junction between putative peptidase and *insL*	23760-23779	KU248944
I-9R	ACATTATCTGACGTGGCTCT			26795-26814	

[a]Sequence used for primer design.

Table S3. Exploration genetic context of the *pco* and *sil* genes by PCR mapping.

Plasmids	GenBank accession no.	PCR								
		A-1	B-2	C-3	D-4	E-5	F-6	J-7	H-8	I-9
pEC5027	KT347600	+	+	+	+	+	+	+	-	-
pS151T	This study	+	+	+	+	+	+	+	-	-
pFS7Z5GT	This study	+	+	+	+	+	+	+	-	-
P3YG7T	This study	+	+	+	+	+	+	+	-	-
pZ13T	This study	+	+	+	+	-	+	+	+	+
pA84T	This study	+	+	+	+	-	+	+	+	+

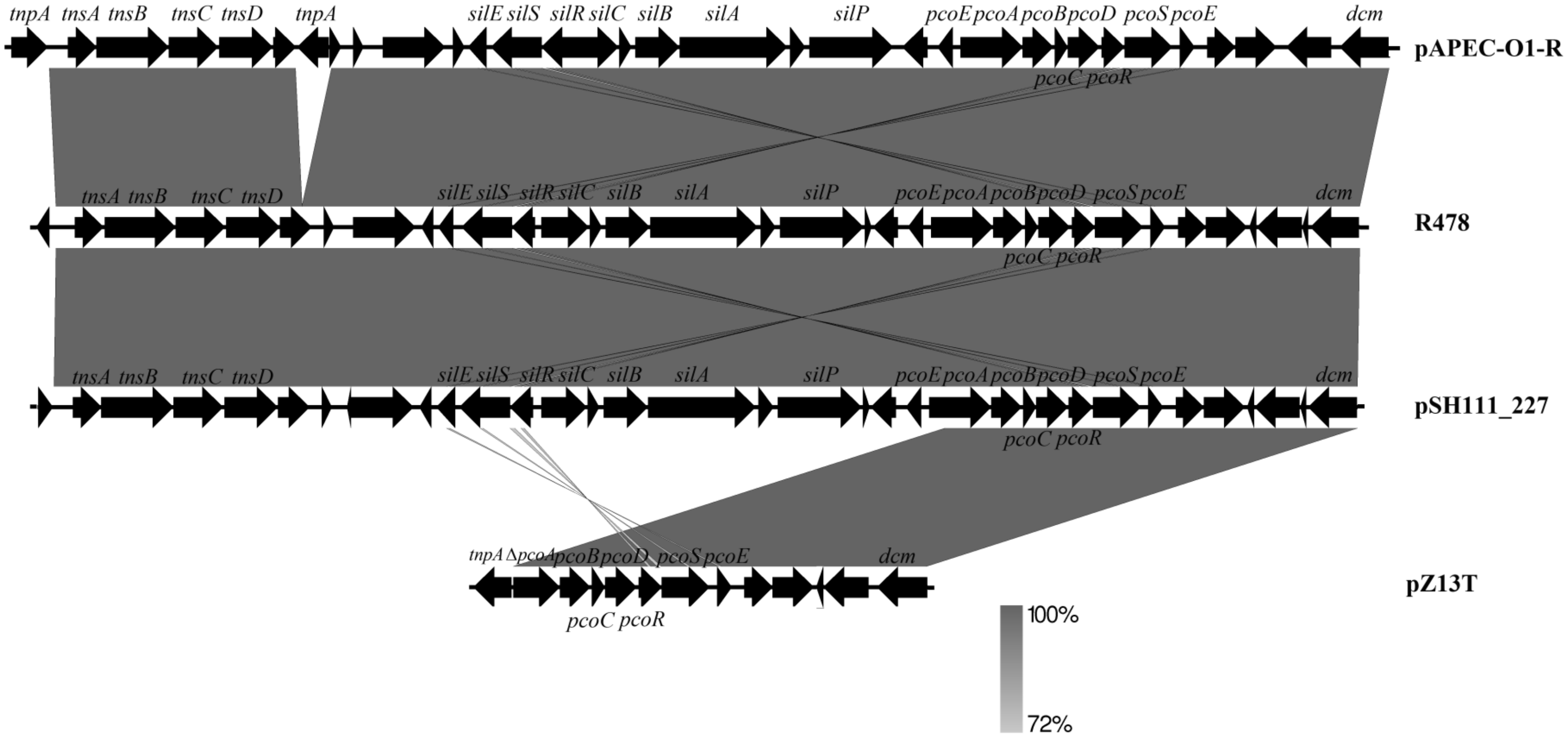

Fig S1 Characteristic of the genetic contexts of the *pco* operons. Plasmids pAPEC-O1-R (BX663045), R478 (DQ517526), and pSH111_227(JN983042) were from GenBank; and pZ13T (KU248943) were from this study.

Fig S2A

Percent Identity

Divergence	1	2	3	4	5	6	7			
1		97.8	95.1	100.0	99.4	100.0	99.4	1	**pAPEC-O1-R**	**(BX663045)**
2	2.2		96.3	97.8	98.3	97.8	98.3	2	**pEC5027**	**(KT347600)**
3	5.0	3.8		95.1	95.1	95.1	95.0	3	**pMG101**	**(AF067954)**
4	0.0	2.2	5.1		99.4	100.0	99.4	4	**pSH111-227**	**(JN983042)**
5	0.6	1.7	5.1	0.6		99.4	100.0	5	**pZ13T**	**(KU248944)**
6	0.0	2.2	5.1	0.0	0.6		99.4	6	**R478**	**(DQ517526)**
7	0.6	1.7	5.1	0.6	0.0	0.6		7	**p3YG7T**	**(KU248945)**
	1	2	3	4	5	6	7			

Fig S2B

Percent Identity

Divergence	1	2	3	4	5	6	7			
1		85.2	86.3	82.6	86.4	86.3	86.3	1	**pEC5027**	**(KT347600)**
2	16.5		99.4	99.4	99.4	99.4	99.4	2	**pRJ1004**	**(X83541)**
3	15.2	0.6		99.9	99.9	99.9	99.9	3	**pSH111_227**	**(JN983042)**
4	19.9	0.6	0.1		100.0	100.0	100.0	4	**pZ13T**	**(KU248943)**
5	15.1	0.6	0.1	0.0		99.9	100.0	5	**R478**	**(DQ517526)**
6	15.2	0.6	0.1	0.0	0.1		99.9	6	**p3YG7T**	**(KU248945)**
7	15.1	0.6	0.1	0.0	0.0	0.1		7	**pAPEC-O1-R**	**(BX663045)**
	1	2	3	4	5	6	7			

Fig S2 DNA sequence alignment of the *sil* or *pco* operons by using the DNASTAR software MagAlign Program. A) Alignment of the nucleotide sequence of the *sil* operon (based on *silESRCBAP* sequence); B) Alignment of the nucleotide sequence of the *pco* operon (based on *pcoEABCDRSE* sequence). The sequence were from plasmids pAPEC-O1-R (BX663045), R478 (DQ517526), pSH111_227(JN983042), pEC5207 (KT347600), pMG101 (AF067954), PRJ1004 (X83541) p3YG7T (this study) and pZ13T (this study).
